# Reliability of an automated gaze‐controlled paradigm for capturing neural responses during visual and face processing in toddlerhood

**DOI:** 10.1002/dev.22157

**Published:** 2021-09-14

**Authors:** Rianne Haartsen, Luke Mason, Eleanor K. Braithwaite, Teresa Del Bianco, Mark H. Johnson, Emily J. H. Jones

**Affiliations:** ^1^ Centre for Brain and Cognitive Development Department of Psychological Sciences, Birkbeck University of London London UK; ^2^ Department of Psychology University of Cambridge Cambridge UK

**Keywords:** child, electroencephalography, evoked potentials, eye‐tracking technology, human development, methods, preschool

## Abstract

Electroencephalography (EEG) has substantial potential value for examining individual differences during early development. Current challenges in developmental EEG research include high dropout rates and low trial numbers, which may in part be due to passive stimulus presentation. Comparability is challenged by idiosyncratic processing pipelines. We present a novel toolbox (“*Braintools”*) that uses gaze‐contingent stimulus presentation and an automated processing pipeline suitable for measuring visual processing through low‐density EEG recordings in the field. We tested the feasibility of this toolbox in 61 2.5‐ to 4‐year olds, and computed test–retest reliability (1‐ to 2‐week interval) of event‐related potentials (ERP) associated with visual (P1) and face processing (N290, P400). Feasibility was good, with 52 toddlers providing some EEG data at the first session. Reliability values for ERP features were moderate when derived from 20 trials; this would allow inclusion of 79% of the 61 toddlers for the P1 and 82% for the N290 and P400. P1 amplitude/latency were more reliable across sessions than for the N290 and P400. Amplitudes were generally more reliable than latencies. Automated and standardized solutions to collection and analysis of event‐related EEG data would allow efficient application in large‐scale global health studies, opening significant potential for examining individual differences in development.

## 1 INTRODUCTION

The human brain develops rapidly during the first 5 years of postnatal life. During this relatively short window of development, a range of cognitive and motor abilities develop and mature from infancy through toddlerhood to preschool (Johnson & De Haan, 2015). Similarly, brain structure and functioning undergo substantial changes (Grayson & Fair, 2017), but our understanding of how these changes in the developing brain are linked to the equally substantive changes in behavioral skills remains very limited. Research into early brain and cognitive development is important scientifically but also has important practical implications for measuring effects of early adversity during development, for example, in global health (Dasgupta et al., 2016; Grantham‐McGregor et al., 2007; Turesky et al., 2019). However, large‐scale efforts to collect neurocognitive data in early development remain rare, in part due to methodological challenges related to scalable measurement of brain functioning in infants and toddlers (Azhari et al., 2020; Brooker et al., 2019; Byers‐Heinlein et al., 2021; Noreika et al., 2020).

One method that can provide temporally sensitive measures of brain functioning is electroencephalography (EEG). Ongoing EEG reflects the synchronized activity of large populations of neurons with a millisecond temporal resolution (Lopes da Silva, 2013). EEG methods are particularly suitable for measuring neural responses in awake infants and toddlers as there are few movement restrictions, in particular when using wireless EEG (Lau‐Zhu et al., 2019). In event‐related approaches, ongoing EEG is time‐locked to stimulus presentation and averaged across multiple trials (Luck, 2014). The resulting sequence of peaks and troughs (event‐related potential [ERP]) represents neural activity reliably elicited by a particular auditory, tactile, or visual stimulus.

Although neural responses to different stimulus categories have been widely studied in the lab, the published literature often disproportionately reflects small groups of infants or young children from high‐income countries (HIC), who may not yield robust, generalizable insights into development and are not suitable for studying individual differences (Henrich et al., 2010). Moving key experimental techniques to field settings where data can be collected at scale is important to ensure greater sample diversity, increased sample size, and greater translation of methodological innovation to population studies. To do this, we must address several challenges. First, traditional ERP paradigms with infants and toddlers are often associated with high dropout rates with a range of 21%–50% (De Haan et al., 2002; Halit et al., 2003; Jones et al., 2016; Stets et al., 2012; van der Velde & Junge, 2020; Webb et al., 2011). Such high attrition rates lead to small sample sizes and constrain analyses of individual differences. For example, analytic methods whose statistical power is affected by cluster size (e.g., random effects models) are often utilized in developmental and global health studies with independent sampling of specific subpopulations (e.g., infants with elevated familial likelihood of autism, or learning disability). Reliable estimates of individual differences with such methods are maximized with appropriate numbers of participants (Austin & Leckie, 2018). In addition, high attrition rates could mean that included samples are biased toward more attentive children, reducing generalizability. Second, to maximize inclusion rates traditional analysis choices for infants and young children use thresholds of 10 trials for inclusion (vs. 20 or 30 in adult studies; Hämmerer et al., 2013; Huffmeijer et al., 2014). Although infant ERP components often show larger amplitudes compared to adults, it is unknown whether a lower number of trials compromises reliability of the measured neural responses. Third, there is low consistency in the selected parameters and manual steps taken during data analyses between different studies, labs, and even experimenters within labs (Noreika et al., 2020). Some automated EEG preprocessing pipelines have been developed, such as the HAPPE pipeline (Gabard‐Durnam et al., 2018), the MARA pipeline (Winkler et al., 2014), the MADE pipeline (Debnath et al., 2020), the EEG‐IP‐L pipeline (Desjardins et al., 2021), and the adjusted ADJUST algorithm (Leach et al., 2020). However, these have been developed for high‐density EEG systems; although high‐density EEG allows assessment of additional metrics like connectivity and source analysis, systems are currently costly and there are few portable versions available that are suitable for use in the field. Large‐scale studies incorporating assessment of single ERP features do not require high‐density systems, and there is thus an additional need for pipelines that are tailored for low‐density/low‐cost EEG (Lau‐Zhu et al., 2019).

The current project tests the feasibility of a fully automated low‐cost approach to collect event‐related EEG data for use in field settings. The project comprises a toolbox—*Braintools—*that utilizes gaze‐contingent stimulus presentation. Gaze‐contingent stimulus presentation allows for data collection paced by the participants themselves, as stimuli are only presented when participants are looking at the screen. With this approach, data collection is tuned to the attentional resources of the participants and should theoretically reduce dropout rates and increase trials numbers and thus data availability. In addition, the *Braintools* toolbox includes scripts for automated harmonized analyses of the data that are not based on visual inspection by researchers (Conte et al., 2020). Braintools is designed for use with a low‐cost, low‐density portable, and wearable EEG system to enable scalability, and thus automated pipelines do not rely on techniques like independent component analysis (ICA) or principle component analysis (PCA) that typically require high‐density arrays (Winkler et al., 2014). The Braintools paradigm has been implemented in studies in both HIC (United Kingdom) and low‐income countries (LIC; India and The Gambia) to examine its potential for global health implementations.

The *Braintools* toolbox includes a range of visual and auditory tasks commonly used in developmental research. Here, we focus on the visual task as visual processing may be a suitable domain for examining individual differences during early development. The rapid development of early visual processing is partly experience dependent, and becomes faster and more efficient with increasing age (Geldart et al., 2002; Röder et al., 2013). The visual cortex develops rapidly during infancy and refines into childhood, supporting the development of visual acuity, contrast sensitivity, and binocularity (Braddick & Atkinson, 2011; Leat et al., 2009; van den Boomen et al., 2015). Visual stimuli can also be readily controlled in experimental designs. Together, these features of visual processing make the domain a good potential marker for examining individual developmental differences.

Event‐related EEG designs can be used to study both early‐stage components associated with domain‐general visual cortical processing, and later‐stage components associated with the development of domain‐specific experience‐dependent expertise. Early stage cortical visual processing is often indexed by the P1, a positive deflection around 100 ms after stimulus onset at occipital electrodes that is associated with low‐level sensory processing of visual stimuli (Rossion & Caharel, 2011) and can be most strongly elicited by high‐contrast and mid‐spatial frequency stimuli such as checkerboards (Benedek et al., 2016). Later‐stage components are more sensitive to more complex higher level processing, such as detecting and discriminating faces. Young infants orient to faces from birth (Johnson et al., 1991), but face processing continues to develop into adolescence (Kilford et al., 2016).

Faces provide important communicative cues that are critical during social communication and interaction (Frith & Frith, 2007). Rapid and efficient face processing, and the ability to extract subtle information from faces is therefore key for social functioning. Event‐related EEG designs are widely used to study face processing, particularly through measuring the face‐sensitive N170 component at parietal electrodes in adults (Bentin et al., 1996). The N290 and the P400 components are thought to represent the infant and toddler precursor of the adult N170 (De Haan et al., 2002; Halit et al., 2003). The N290 is a negative deflection around 290 ms after stimulus onset, while the P400 is a positive deflection around 400 ms after stimulus onset. The N290 shows a faster latency and larger amplitude (more negative) response to face stimuli than nonface stimuli, like cars, objects, and houses (Kuefner et al., 2010). The P400 shows a smaller amplitude (less positive) for faces than nonface stimuli (Conte et al., 2020; Di Lorenzo et al., 2020; Jones et al., 2017). Furthermore, the N290 amplitude is larger for inverted than upright human faces, while the N290 latencies and P400 amplitudes are similar for both orientations in 12‐month‐old infants (De Haan et al., 2002). Toddlers, however, show a larger P400 amplitude for inverted compared to upright faces (Peykarjou et al., 2013), whereas the N290/N170 amplitude and latency show no modulations by orientation (Henderson et al., 2003; Peykarjou et al., 2013). Children and adolescents show longer N170 latencies for inverted than upright faces, while the inversion effect on N170 amplitude increases with age showing more negative amplitudes for inverted faces (Itier & Taylor, 2004b). The N290 and P400 have often been the measures of interest in studies examining developmental trajectories in young typically developing children and young children with developmental disorders (Bhavnani et al., 2021).

Although studies using EEG to examine categorical response differences in groups of infants and toddlers have provided clear insights into brain development, using EEG measures to assess individual differences brings additional challenges. Research into individual differences requires robust signals at an individual level that are stable and reliable over brief time windows. Such measures could help track early development of visual processing in relation to early adversity, for example, in large population studies or in the context of global health research. Encouragingly, previous studies have found test–retest reliabilities in typically developing infants at values of 0.76 for N290 mean amplitude, and 0.56 for P400 mean amplitude in response to faces over a 1‐ to 2‐week interval (Munsters et al., 2019), and in typically developing 6‐ to 12‐year‐old children at values of 0.80 for P1 peak amplitude in response to checkerboards and 0.77 for N170 peak amplitude in response to faces over a 6‐ to 8‐week interval (Webb et al., 2020). However, similar values for toddlers are not available as this has traditionally been an age range in which data are very difficult to collect. Early event‐related measures may have the potential to help identify young children who show atypical developmental trajectories and to predict later outcomes. For instance, individual variability in face processing in young children may predict later behavioral outcomes during toddlerhood and mid‐childhood, such as autism (Elsabbagh et al., 2009; Shephard et al., 2020). To examine individual differences in visual processing across early development in a reliable and robust way, there is a need for novel methods that reduce dropout rates, are reliable, increase data availability, and standardize data analyses.

Here, we examined the feasibility and test–retest reliability of the *Braintools* toolbox in typically developing 2.5‐ to 4‐year‐old toddlers in the United Kingdom as a first step. We examined P1 components to checkerboards, and N290 and P400 components to faces (upright and inverted orientation). We focused on early childhood because this age range can be the most challenging to test (Brooker et al., 2019); a recent review noted there have been no reliability studies during toddlerhood (Brooker et al., 2019); and this is the age range in which many neurodevelopmental disorders become initially apparent in behavior, making it an important age range for large‐scale studies of child development. Toddlers were tested twice over a 1‐ to 2‐week interval with a low‐density EEG system. We chose 1–2 weeks as the interval because shorter intervals might lead to repetition effects in the neural responses and data loss. Young children are less interested in stimuli that they have recently seen. This results in lower numbers of artifact‐free trials and subsequently into smaller sample sizes for test–retest reliability analyses (Haartsen et al., 2020). In contrast, longer intervals may reflect developmental change rather than stability of the measures (Blasi et al., 2014; Haartsen et al., 2020). We estimated the reliability of measures of P1, N290 and P400 peak latency, peak amplitude, and mean amplitude. As both previous research with infants (Munsters et al., 2019) and adults (Huffmeijer et al., 2014) suggests peak latency measures are not robust, we additionally examined the utility of a method called “dynamic time warping” (DTW), which rather than relying on the identification of individual peaks, warps an individual EEG signal until it matches a reference signal to identify the general delay between the two (Zoumpoulaki et al., 2015). We computed intraclass correlations (ICCs) of the ERP measures across different numbers of included trials in order to make recommendations about the minimum trial numbers needed for stable estimates. We furthermore calculated ICCs within sessions or split‐half reliability to examine internal stability at different numbers of trials.

## 2 METHODS

### 2.1 Participants

Sixty‐one (34 female) typically developing full‐term children were recruited when they were between 30‐ and 48‐month old from the Greater London area via the Birkbeck Babylab database. Participants were invited to attend two visits to the lab with an interval of 1–2 weeks between visits. Parents/caregivers gave written informed consent upon arrival at the Babylab and after the study was explained to them by the researchers. They also filled out a demographic questionnaire, a medical questionnaire, and a language questionnaire.

The group of children in this whole recruited sample had an average age of 38.36 months (SD = 4.67, ranging from 30 to 49 months) at the day of their first visit. The second visit took place for 51 of these children with an average 10 days after the first visit (SD = 5, ranging from 7 to 28 days). Annual household income for the families was below £40,000 for 25% of the children in the recruited sample, between £40,000 and £99,999 for 44%, and above £100,000 for 32% (data were missing for *N* = 4). Households on average consisted of 1.47 people per bedroom (SD = 0.47), ranging from 0.75 to 3 people per bedroom (data missing for *N* = 3). Further, 30 of 61 children heard English at least 95% of the time at both home and nursery (data missing for *N* = 8). Other languages the children heard were Amharic, French, Gujarati, German, Hindu/ Urdu, Italian, Malayalam, Mandarin, Romanian, Russian, Polish, Portuguese (also Brazilian Portuguese), Spanish, and Turkish. Finally, four children had speech delays requiring speech therapy, and two children had a relative with speech delays requiring speech therapy.

Participants received a Babylab tote bag or t‐shirt and certificate after completing the study. This study received ethical approval from the Department of Psychological Sciences ethics committee at Birkbeck (ref. no. 171874).

### 2.2 Stimuli and apparatus

**FIGURE 1:**
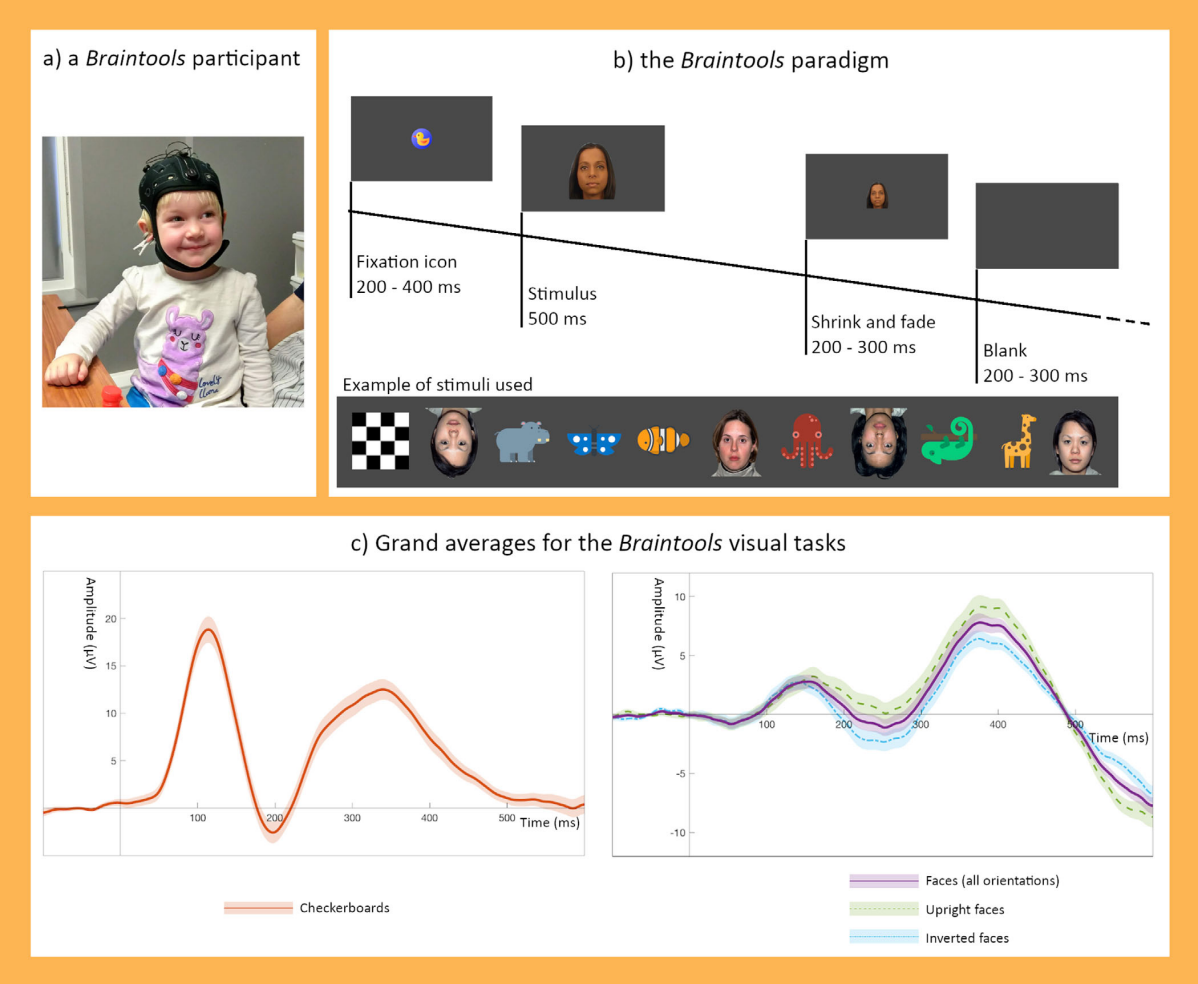
The *Braintools* paradigm. Children participated in the Braintools study wearing an Enobio EEG system (a). While their EEG was recorded, they watched the *FastERP* task where checkerboards, faces, and animals being presented in blocks of trials (b). The grand averages across sessions for the checkerboard trials (c left) and the face trials (c right) display clear peaks for the ERP components: P1 during checkerboards, and N290 and P400 during faces. Shaded areas around the grand averages represent the standard error of the mean across sessions

At each session, children participated in a 25‐min EEG battery. Depending on their engagement, the visual and auditory battery duration varied between 22 and 27 min. The first portion of the EEG battery included the FastERP task, interspersed with short cartoon clips and dynamic videos. At the end of the battery, children participated in an auditory oddball task. The FastERP task consisted of the presentation of visual stimuli and recording the event‐related response (Figure 1).

The visual stimuli presented on each trial were either one of four female faces (of African, Asian, South Asian, and Caucasian ethnicity; Tye et al., 2013), one of the 50 cartoon images of animals (designed by Freepik from Flaticon), or a checkerboard (Benedek et al., 2016). Examples of the stimuli are presented in Figure 1b. Both faces and animals were presented in upright and inverted (180° rotated) orientation. In total, 144 trials with faces (18 upright and 18 inverted repeated presentations for each of the four faces), 72 trials with animals (36 upright and 36 inverted images randomly selected of 50 possible cartoon images), and 72 trials with the checkerboard were presented in randomized order within 18 blocks of 16 trials each, with a constraint that no more than three of the same stimulus types (face/animal/checkerboard) were presented in a row. Each block began with a gaze‐contingent fixation stimulus (Flaticon image of an object, 3 by 3 cm with a subtended visual angle of 2.9°), which was randomly selected. When participants fixated the Flaticon, after a random 200–400 ms delay the Flaticon was replaced by the stimulus (animal or checkerboard; both width and height of 17 cm with a subtended visual angle of 16.1°; or a face with width of 17 cm and height of 21.8 cm with a subtended visual angle of 20.6°), presented for 500 ms. The stimulus then shrank to a size of 0.2 cm (over a random 200–300 ms period) and faded out. After a blank screen (200–300 ms variable), either another stimulus was presented, or a fixation stimulus if the participant had looked away from the screen. Cheerful instrumental music was continuously played during the FastERP task.

Blocks of the FastERP task were alternated with dynamic video clips and short cartoon clips (not analyzed here). The dynamic videos consisted of a social video and a nonsocial video f which presentation was alternated (Jones et al., 2015). The social video consisted of five short vignettes of two women singing nursery rhymes with a total duration of 60 s. The nonsocial video consisted of six short vignettes of spinning and moving toys with a total duration of 60 s. The 12 short cartoon clips displayed animals and had a duration of ∼5 s. Each session started with the presentation of the dynamic social video, followed by a block of the FastERP task. The FastERP task and video clips were alternated during the rest of the session. Each dynamic video clip was presented three times, and each animal cartoon clip was presented once. Presentation order of the FastERP task, dynamic videos, and animal cartoon clips was identical for all participants.

Stimulus presentation was controlled by a MacBook Pro (15‐inch, part number MR942B/A, with an eighth‐generation Intel i7 6‐core processor, 2016) on an external monitor screen (Asus VG248, 24‐inch screen size, 1920 × 1080 resolution at 60 Hz). A portable Tobii Pro X2‐60 eye‐tracker was attached below the screen and data were recorded with Tobii Pro SDK 3Manager. Stimuli were presented and data were saved from the laptop using the stimulus presentation framework, Task Engine (https://sites.google.com/site/taskenginedoc/; Jones et al., 2019). This framework is optimized for standardized EEG and eye‐tracking (ET) data collection. Here, the framework was run on a macOS High Sierra 10.13.6 system, in Matlab R2017a, with Psychtoolbox 3.0.14, Gstreamer 1.14.2 for stimulus presentation, and a Lab Streaming Layer to connect to the EEG system; EEG and ET task events were time stamped and saved in Matlab format.

EEG was simultaneously recorded with a wireless geltrode Enobio EEG system (NE Neuroelectrics, Barcelona, Spain) and transmitted to the laptop using Bluetooth. Eight EEG electrodes were placed at FPz, Fz, Cz, Oz, C3, C4, P7, and P8 (also see Supplementary Materials SM1 for channel layout). We selected a low‐density array because for EEG with children with hair (i.e., not babies), the amount of time required to appropriately seat electrodes and ensure a good quality signal scales with the number of electrodes. Larger arrays can thus increase dropout rates for children with limited patience for cap placement, and also can be challenging to incorporate in large‐scale studies where time is essential. Given our focus on selected ERP signals measured at known locations, we were able to utilize a low‐density array focused on the key locations of interest: Oz on the occipital area to measure the P1 component (Benedek et al., 2016; Rossion & Caharel, 2011), and P7 and P8 over parietal areas to measure the N290 and P400 components (Conte et al., 2020; De Haan et al., 2002; Di Lorenzo et al., 2020; Halit et al., 2003; Jones et al., 2017).

The CMS and DRL electrodes were attached to a clip on the participants’ ear. For participants who refused to wear the ear clip, the CMS and DRL electrodes were placed on the mastoid with stickers (*N* = 8). Data were recorded with a 500 Hz sampling rate using Neuroelectrics NIC 2.0 software (Barcelona, Spain). Data quality during the session was monitored using the Neuroelectrics Quality Index (QI) rather than impedance check (impedance check feature is not incorporated in the EEG system used here). The QI is calculated from line noise (power in 49–51 Hz range), main noise (power in the 1–40 Hz frequency range), and the offset of the signal every 2 s. NIC software shows the color codes of the QI for each channel with green for low QI and good data, orange average data quality, and red for high QI and low data quality.

Each session was recorded with a webcam (HD Pro Webcam C920) and Open Broadcaster Software (OBS).

### 2.3 Procedure

Procedures were identical for both sessions and were performed by two researchers per session (EB, TDB). During the experiment, children were seated on their parent or caregiver's lap at approximately 60 cm from the screen. The EEG cap and ear clip were positioned on the children while they watched a cartoon video of their choice until all of the EEG signals were of sufficient quality. The researchers aimed to improve data quality until each of the QI values for the EEG channels were green (or most green and some orange depending on the state and tolerance of the participant). Parents/caregivers were asked to wear a pair of plastic shutter glasses to ensure the eye‐tracker picked up only the child's eye gaze. After a successful five‐point ET calibration, the experimenters proceeded with the Braintools battery (or if at least four of five points were successfully calibrated, defined per calibration point as accuracy and precision both less than 2.5° of visual angle from at least one eye). During this battery, the visual task was presented in blocks, and music was played throughout the session in order to keep the children engaged. Researchers further attempted to improve the signal quality when they noticed EEG quality dropped (by inspection of the EEG signal or red color codes).

Blocks and trials for the visual task were presented in a gaze contingent fashion; trials were only presented when the children were looking at the screen and presentation paused when the children looked away. Researchers asked the toddlers to name or count the animals they were seeing out loud. If the children were not looking at the screen for a prolonged period of time, experimenters tried to re‐engage the children's attention to the screen by playing a short auditory attention getter.

### 2.4 Assessment of visual attention

Gaze was extracted from the ET recording during the presentation of the stimulus for each trial (0–500 ms). We interpolated gaps with a duration of 150 ms or less and then calculated the number of valid trials for the FastERP task. A trial was considered valid if the proportional looking time during the stimulus presentation was 50% or above. We then calculated the percentage of valid ET trials relative to the total number of presented trials in the ET data as a measure of attentiveness during the whole session. Based on these data, we created a subsample of children who were very attentive (≥60% trials attended for both sessions) within which to additionally assess reliability.

### 2.5 Data preprocessing

Data were preprocessed using a combination of in‐house written scripts and Fieldtrip Matlab scripts in Matlab R2018b (in‐house scripts are available on GitHub; Haartsen et al., 2021, and Fieldtrip scripts are available via; Oostenveld et al., 2011). First, data validity was checked and corrected where necessary and possible, for example, cases with technical issues during data collection or saving, or inaccurate timing or absence of EEG markers. Enobio data were converted into Fieldtrip format for further preprocessing.

Continuous data were segmented into trials from −100 to 600 ms after stimulus onset. Then trials were split into two datasets by condition (checkerboards or faces) in order to apply task‐specific processing steps. For the checkerboard trials, signals for the occipital channel Oz, and C3, C4, and Cz (for later re‐referencing) were filtered using a 0.1–40 Hz bandpass filter (with 3 s padding on both sides) to filter out high‐frequency noise from muscle artifacts and a dft filter (at 50, 100, and 150 Hz) to filter out the still remaining residual line noise due to the shallower slope of the bandpass filter. Data were baseline corrected with −100 to 0 as baseline window. Trials were marked bad if the signal was flat (if amplitude did not exceed 0.0001 µV) or exceeded thresholds of −150 and 150 µV (selected based on initial inspection of data quality at different thresholds, see SM2). A channel was excluded if artifacts were present for 80% of the trials or above and a trial was excluded if the signal for Oz included artifacts. Next, we re‐referenced the data on a trial‐to‐trial basis to Cz, or the average of C3 and C4 if Cz contained artifacts. If channels C3 and/or C4 contained artifacts as well, the whole trial was excluded from further analysis.

For the face trials (upright and inverted, all ethnicities), we selected parietal channels P7 and P8, and Cz, C3, and C4 for later re‐referencing. Filters, baseline correction and artifact identification were identical to those used for the checkerboard trials. A channel was excluded if the signal was bad for 80% of the trials or above and a trial was excluded if the signal for P7 and/or P8 contained artifacts. Finally, the re‐referencing procedure for face trials was identical to the procedure for the checkerboard trials.

The animal trials were intended to maintain toddler attention in the paradigm. For completeness, we preprocessed these trials with the same parameters as the face trials. Further visual inspection of the grand averages of the animal trials indicated that the event‐related responses elicited were weak (see SM3). These trials may not provide comparable neural responses because toddlers sometimes named or counted the animals; and because the icons chosen had lower visual complexity than the faces. We therefore excluded the animal trials from further analyses.

### 2.6 Extracting ERP features

**FIGURE 2:**
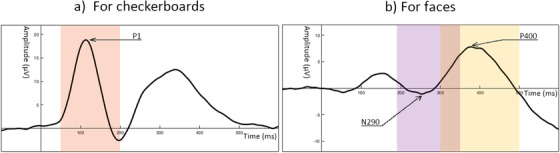
Time windows of interest for the components of interest. Grand average during checkerboards with the P1 peak (arrow) and time window of interest (50–200 ms in orange) is displayed on the left (a), and grand average during faces (all orientations) with the N290 and P400 peak (arrow) and time windows of interest (N290: 190–350 ms in purple, and P400: 300–500 ms in yellow) is displayed on the right (b)

ERP components are typically defined as peaks within prespecified time windows. We based time windows of interest on both previous literature and grand averages of the current data (see Figure 1). Time windows for the P1 component in previous studies were 90–170 ms in 4‐year olds (van den Boomen et al., 2015), and 50–200 ms in 4‐year olds (Jones et al., 2018). The N290 was identified within windows of 286–610 ms in 3‐ to 4‐year olds (Dawson et al., 2002), and 190–390 ms in 4‐year olds (Jones et al., 2018), whereas the P400 was identified within windows of 286–610 ms in 3‐ to 4‐year olds (Dawson et al., 2002), and 300–600 ms in 4‐year olds (Jones et al., 2018). We adjusted these time windows for our age and setting by examining grand averages of the components across all available data sessions with 10 artifact‐free trials or more (note; we included both test and retest datasets and used a cutoff of 10 trials in accordance with previous ERP studies; Jones et al., 2018; Webb et al., 2011). P1 was measured at Oz, and N290/P400 at the average of the P7 and P8. As an additional check for individual averages with limited signal content, we excluded individually averaged time series from the grand average where amplitudes did not exceed 1 or −1 µV. We then confirmed that the peaks in the grand averages were within the peaks from the previous studies. We adjusted the end of the N290 and start of the P400 window to decrease the overlap, and the end of the P400 window to match up with the offset of the stimulus. Final windows were: P1—most positive peak between 50 and 200 ms after stimulus onset; N290—most negative peak between 190 and 350 ms after stimulus onset; P400—between 300 and 500 ms after stimulus onset (see Figure 2).

To examine test–retest reliability across different numbers of trials, we randomly selected subsets of trials from the clean datasets for each individual: (a) 10–20–30–40–50—all available trials for checkerboards, (b) 10–20–30–40–50–60–70–80–90–100—all available trials for faces (where an equal balance of upright and inverted were used), and (c) 10–20–30–40–50–60—all available trials for faces upright and faces inverted separately. We averaged EEG data across these randomly selected trials (and channels of interest if multiple) for checkerboards and faces and applied a baseline correction (−100 to 0 ms as baseline) to the time series.

Peaks for the individually averaged ERPs were identified with a peak identification algorithm. First, all positive peaks were identified by testing at each EEG sample (sample *X*) if the amplitude was *larger* than the amplitude of following EEG sample (sample *X* + 1). If the following sample (sample *X* + 1) had the *same* amplitude as the current sample (sample *X*), the algorithm tested whether the sample after (sample *X* + 2) had a *lower* amplitude (negative deflection) and identified the following sample (sample *X* + 1) as a *positive* peak. If the following sample (sample *X* + 1) had a *smaller* amplitude than the next sample (sample *X* + 2), this sample was considered as part of a plateau and the algorithm skipped to the next sample. Second, all negative peaks were identified by testing at each EEG sample (sample *X*) if the amplitude was *smaller* than the amplitude of the following sample (sample *X* + 1). If the following sample (sample *X* + 1) had the *same* amplitude as the current sample (sample *X*), the algorithm tested whether the sample after (sample *X* + 2) had a *higher* amplitude (positive deflection) and identified the following sample (sample *X* + 1) as a *negative* peak. If the following sample (sample *X* + 1) had a *larger* amplitude than the next sample (sample *X* + 2), this sample was considered as part of a plateau and the algorithm skipped to the next sample. The result of this algorithm was a list of all positive and negative peaks identified throughout the ERP waveform.

From this list of peaks, the peaks of interest were selected. For the P1 peak, all positive peaks were selected occurring in our time window of interest (50–200 ms). We selected the identified peak as P1 peak if there was only one peak within the window. If multiple positive peaks were present during the time window, we selected the peak with the largest amplitude as the P1 peak. If no positive peaks were present in the time window, we widened our window with 20 ms on either side (30–220 ms) and selected the only peak or largest peak if multiple peaks were present. In the instances where no peaks were found in the wide window either, we noted down that no P1 peak was identified and the P1 was considered invalid. These waveforms were excluded from further analyses (to ensure sample sizes for reliability analyses were consistent across P1 features).

For the N290 peak, we selected all negative peaks occurring during our time window of interest (190–200 ms). As for the P1 peak selection, we selected the negative peak as N290 peak if only one peak was present during the time window. Otherwise, we selected the negative peak with the largest amplitude. If no negative peak was present, we widened our window with 20 ms on either side (170–370 ms). The only peak or peak with largest amplitude was selected as N290 peak. If no negative peaks were identified in this process, we considered this N290 as invalid and the waveform was excluded from further analyses.

For checkerboards, we extracted P1 peak latency and peak amplitude (averaged across a 60‐ms window centered around the P1 peak by the algorithm described above and if valid). For faces, we extracted N290 peak latency, peak amplitude (average amplitude across a 60‐ms window centered around the N290 peak by the algorithm described above and if valid) and mean amplitude (190–350 ms). We also calculated P400 mean amplitude (300–500 ms); we did not compute peak latency or amplitude because the P400 has a wider peak morphology and its peak is consequently more difficult to identify. Identified peaks were considered likely to represent “noise” and removed from further analysis if the amplitude at the ERP peak latency did not exceed the amplitude of the largest peak of the same directionality (positive or negative) during the baseline. Note, amplitudes here were calculated to the point of the peak instead of averaged across a 60‐ms window centered around the peak for the ERP component features. With this method we avoided averaging across 60% of our baseline window (−100 to 0 ms) and averaging across samples outside the baseline window (if peak was close to −100 ms) or during the presentation of the stimulus (if the peak was close to 0 ms) as would be the case with averaging across a 60‐ms window centered around the peak in the baseline window. These measures were extracted across ERPs for faces collapsed, faces upright, and faces inverted.

Another way to extract the latency of neural responses is to apply DTW to the data. This method takes an individual waveform and calculates the distance it needs to be transformed or warped in order to resemble a reference waveform. The result of this calculation is a warping path with distances between two waveforms across the time series. The DTW direction measure is defined as the area between this warping path and the main diagonal of the cost matrix normalized by area under the diagonal (also termed *DTW_diff_* in Zoumpoulaki et al., 2015). A DTW direction value of 0 would indicate the individual waveform and reference waveform are identical. A negative value would reflect a shorter latency in the individual waveform than the reference waveform, whereas a positive value would reflect a longer latency in the individual waveform than the reference waveform. This method provides a general measure of neural processing speed across a whole waveform or time window. Considering the challenges with peak identification in younger children, this method may be more reliable and more robust than traditional peak identification. In the current study, we use the grand average as a reference waveform and examine the DTW direction between the reference waveform and the individual waveform based on different numbers of trials. We calculated DTW direction for checkerboard waveforms during the P1 time window (50–200 ms) and for face waveforms (collapsed, upright, and inverted faces) during the N290 time window (190–350 ms).

### 2.7 Statistical analyses

To assess the feasibility of the paradigm, we calculated the percentage of included participants for analyses focused on visual processing and face processing relative to both the full recruited sample; and relative to the sample who had data from at least one EEG session. We report both percentages for completeness to reflect retention rates when including and excluding participants whose sessions had technical issues or where participants refused to wear the EEG cap.

Next, we tested the face processing condition effects in our sample of toddlers with a first EEG session. We performed a paired *t*‐test comparing ERP features between upright faces and inverted faces (N290 peak latency, peak amplitude, and mean amplitude, P400 mean amplitude, and DTW direction during the N290 time window). We included toddlers with 20 or more artifact‐free trials for each condition.

We assessed test–retest reliability of the ERP features using ICC between measures at visit 1 and visit 2 as in Haartsen et al. (2020) and van der Velde et al. (2019). We used the ICC(3,1) that is a two‐way fixed model ICC suitable to measure the consistency between single scores (Salarian, 2016; Shrout & Fleiss, 1979; Weir, 2005). The ICC is calculated as:

$$ICC\left(3,1\right)=\frac{MS_{R}-MS_{E}}{MS_{R}+\left(k-1\right)MS_{E}}.$$

In this formula, *MS_R_* is the variance between objects (here participants), *MS_E_* is the error variability (also mean squared error), while *k* is the number of measurements per participant (here 2 as there are two sessions). Values for the ICC typically range between 0 and 1, with values close to 0 indicating poor test–retest reliability, and those close to 1 indicating excellent reliability. We interpreted the ICC values in accordance with the following convention: ICC values below .40 as poor; ICC values from .40 to .59 as fair, ICC values from .60 to .75 as good, and ICCs above .75 as excellent (Haartsen et al., 2020; Hardmeier et al., 2014; van der Velde et al., 2019). ICC values were calculated for ERP features of waveforms for the checkerboards, faces (collapsed across condition to increase trial numbers), and the face inversion effects. For the latter, we calculated the face inversion effect subtracting the ERP features for faces inverted from the ERP features for the upright faces at each visit. We then calculated the reliability of this inversion effect between visit 1 and visit 2.

## 3 RESULTS

### 3.1 Feasibility of the paradigm

**FIGURE 3:**
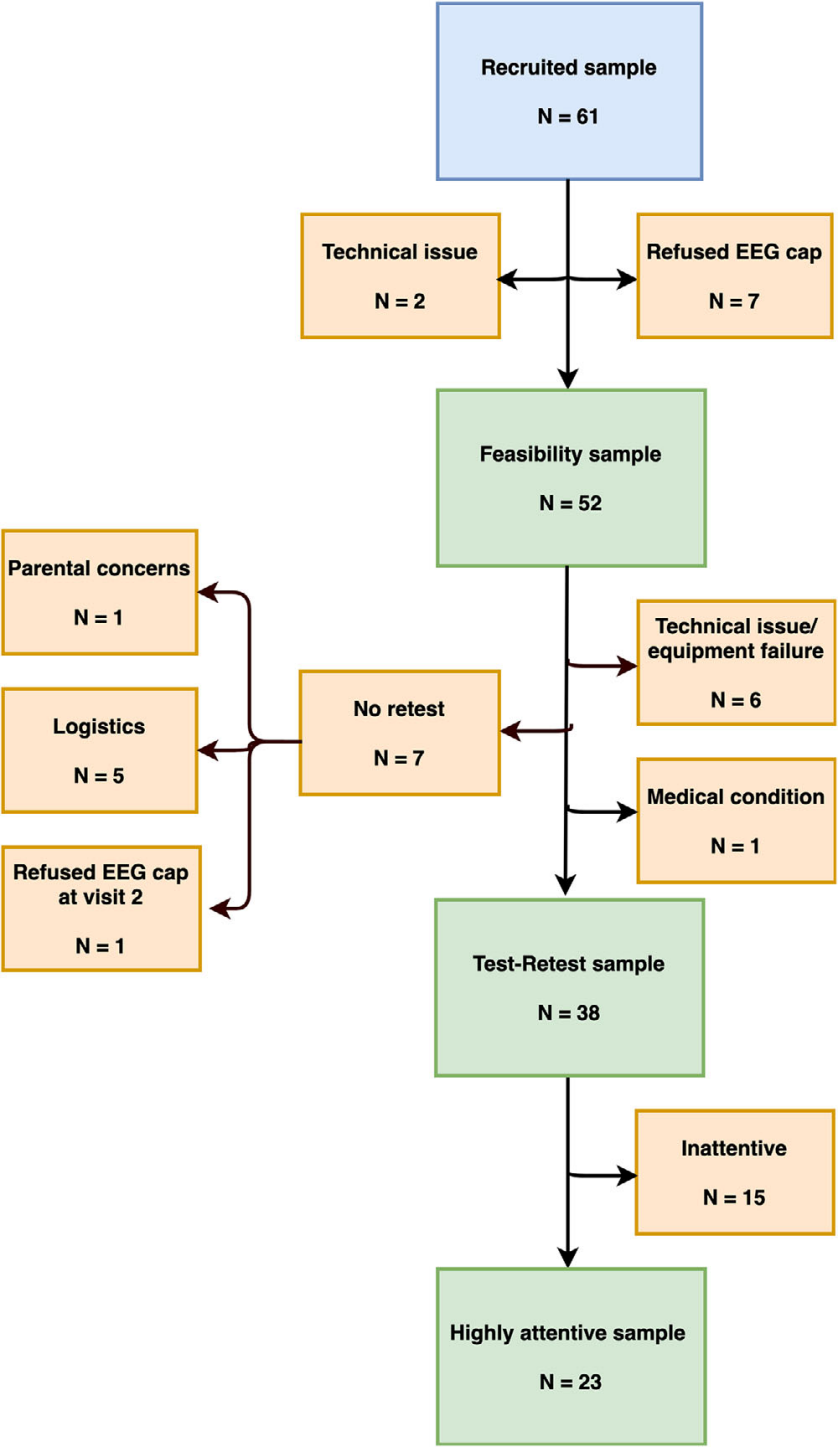
Flowchart of the subsamples. Numbers of participants in the recruited, feasibility, included (test–retest), and highly attentive sample and reasons for exclusion

In total, 61 toddlers were invited to take part in the study consisting of two sessions (hereafter named the recruited sample, also see flowchart in Figure 3). Fifty‐two toddlers provided data for their first session without technical issues (e.g., computer issues—in this case, the second session with the toddler was counted toward this total) (hereafter named the feasibility sample). We were not able to collect data from a first session for nine toddlers (due to cap refusal at the first session and not being invited back for a second session [*N* = 7] and technical issues at both sessions [*N* = 2]).

For the checkerboards, 84% of the toddlers in the recruited sample and 98% of the feasibility sample had at least one trial with clean EEG data (see Table 1). Of the recruited sample, 80% had a minimum of 10 artifact‐free trials, and 79% and 75% had a minimum of 20 and 30 trials. The highest dropout rate was for a cutoff of 50 trials where only 64% of the sample would be included. The inclusion percentages for the feasibility sample were 94%, 92%, 88%, and 75% with a cutoff of 10, 20, 30, and 50 trials, respectively.

**TABLE 1:** Percentages of included participants for different trial cutoffs

Visual processing—Checkerboards			Face processing—Faces			Face processing—Face inversion effect		
Minimum *N* trials	Percentage included relative to the recruited sample	Percentage included relative to the feasibility sample	Minimum *N* trials	Percentage included relative to the recruited sample	Percentage included relative to the feasibility sample	Minimum *N* trials for both conditions	Percentage included relative to the recruited sample	Percentage included relative to the feasibility sample
1	84%	98%	1	84%	98%	1	84%	98%
10	80%	94%	10	84%	98%	10	82%	96%
20	79%	92%	20	82%	96%	20	77%	90%
30	75%	88%	30	82%	96%	30	72%	85%
40	69%	80%	40	79%	92%	40	66%	77%
50	64%	75%	50	75%	88%	50	61%	71%
			60	74%	87%	60	43%	50%
			70	70%	83%	70	3%	4%
			80	67%	79%			
			90	66%	77%			
			100	62%	73%			

For the faces, 84% of the recruited sample had at least 1 or 10 artifact‐free EEG trials. Overall, 82% of the recruited toddlers would be included with a cutoff of 20 or 30 trials. At a cutoff of 100 trials, 62% of the recruited sample would be included in any main analyses. The inclusion percentages for the feasibility sample were 98%, 96%, and 73% with a cutoff of 1 or 10, 20 or 30, and 100 trials, respectively.

For the inversion effect in faces, 84% and 98% of the recruited and feasibility sample, respectively, had more than one trial for both face conditions, and 82% and 96% of the samples could be included with a cutoff of 10 trials. For higher cutoffs of 20, 30, and 40 trials, the inclusion percentages for the recruited sample were as 77%, 72%, and 66%, respectively, and for the feasibility sample these were 90%, 85%, and 77%.

In our feasibility sample, the face inversion revealed a main effect for P400 mean amplitude (*t*(46) = −2.91, *p* = .006), where the P400 amplitude was higher for inverted faces (mean = 4.46 µV, SD = 5.13) than upright faces (mean = 2.92 µV, SD = 3.86). No face inversion effects were observed for the other ERP features (range: .100 ≤ *p's* ≤ .282).

### 3.2 Reliability of ERP measures

For our reliability analyses, we included children with data at both test and retest sessions. Our final included sample for the test–retest reliability analyses consisted of 38 children with clean EEG trials at both test and retest sessions. Of the 52 toddlers with data at either session, we excluded 14 children for the following reasons (see flowchart in Figure 3): families did not return for a second visit due to logistical issues (*N* = 5) or parental concern (*N* = 1), the child refused the cap at their second visit only (*N* = 1), technical issues and equipment failure at one visit (*N* = 6), and a medical condition (*N* = 1).

Overall, 23 of 38 children attended to 60% or more of the presented trials during both test and retest sessions. To explore whether EEG metrics were more reliable within highly attentive children (most likely to be included in previous studies with high attrition rates) we ran the EEG reliability analyses on both the full included sample of 38 children and the highly attentive sample of 23 children. However, it is important to note that there was no clear correlation between the number of clean EEG trials at the test and the retest session in this group (*r*_checkerboard_ = −.05, *p* = .822, and *r*_faces_ = −.06, *p* = .770), nor in the included sample (*r*_checkerboard_ = −.16, *p* = .352, and *r*_faces_ = −.15, *p* = .374). This indicates that general attentiveness or data quantity is not likely to be a stable measure of individual differences in this age range, making it less likely to confound the generalizability of reliability metrics.

Demographics for the recruited, feasibility, included, and highly attentive samples are displayed in Table 2. In the included sample, 25 of 38 children heard English at least 95% of the time at both home and nursery, and two children had speech delays requiring speech therapy. In the highly attentive sample, 15 of 23 children heard English at least 95% of the time at both home and nursery, and one child had speech delays requiring speech therapy.

**TABLE 2:** Descriptive data for (i) the whole recruited sample, (ii) the feasibility sample, (iii) the included sample, and (iv) the highly attentive sample

	Whole sample	Feasibility sample	Included sample	Highly attentive sample
Number of participants (females)	61 (34)	52 (27)	38 (20)	23 (15)
Participant age at first visit (months)	38.36 (4.67), 30–49	38.19 (4.84), 30–49	37.71 (4.56), 30–49	38.22 (4.99), 32–49
Time between visits (days)^a^	10 (5), 7–28	10 (5), 7–28	10 (5), 7–28	10 (4), 7–21
Household income^b^				
<£20,000	7%	6.00%	5.60%	4.50%
£20,000–£29,999	8.80%	10.00%	11.10%	13.60%
£30,000–£39,999	8.80%	8.00%	5.60%	0%
£40,000–£59,999	12.30%	12.00%	16.70%	13.60%
£60,000–£79,999	10.50%	12.00%	11.10%	9.10%
£80,000–£99,999	21.10%	22.00%	27.80%	40.90%
£100,000–£149,999	24.60%	24.00%	13.90%	4.50%
>£149,999	7%	6.00%	8.30%	13.60%

*Note*: Mean (SD), minimum–maximum.

^a^ Data missing for 10 participants in the whole sample.

^b^ Data missing for four participants in whole sample, two participants in the included sample, and for one participant in the highly attentive sample.

#### 3.2.1 Test–retest reliability of ERPs during low‐level visual processing

The ICC values for measures during low‐level visual processing of the checkerboards for the included and highly attentive sample are displayed in Table 3. In the included sample, ICC values were within the poor or fair range for P1 measures. Values were fair for P1 peak latency and peak amplitude for across 20 and 30 trials (ICC_latency_ = .51 and .41 for 20 and 30 trials, and ICC_peak amplitude_ = .46 and .44 for 20 and 30 trials), and even good for peak amplitude across 50 trials (ICC = .62). Values for DTW during the P1 time window were good for 30 trials (ICC = .64). The pattern of ICC values in the highly attentive sample was very similar to the pattern in the included sample.

**TABLE 3:** ICC values for EEG key metrics during low‐level visual processing

		Included sample											
		P1 peak latency				P1 peak amplitude				DTW during P1 window			
Individual ERP based on *N* trials	Number of subjects	ICC	LB	UB	*p*‐Value	ICC	LB	UB	*p*‐Value	ICC	LB	UB	*p*‐Value
10	31	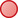 **.38**	.03	.64	**.016**	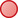 **.36**	.01	.63	**.022**	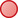 **.31**	−.05	.59	**.044**
20	29	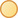 **.51**	.18	.73	**.002**	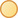 **.46**	.12	.70	**.005**	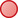 .16	−.21	.49	.201
30	27	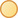 **.41**	.05	.68	**.015**	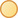 **.44**	.08	.70	**.009**	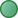 **.64**	.35	.82	**.000**
40	26	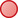 **.33**	−.05	.63	**.044**	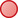 **.35**	−.04	.64	**.037**	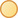 **.46**	.09	.71	**.008**
50	21	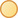 **.42**	.00	.72	**.026**	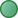 **.62**	.26	.82	**.001**	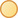 **.51**	.11	.77	**.008**
all	36	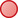 .23	−.10	.51	.087	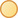 **.57**	.30	.75	**.000**	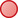 **.29**	−.03	.56	**.039**

		Highly attentive sample											
		P1 peak latency				P1 peak amplitude				DTW during P1 window			
Individual ERP based on *N* trials	Number of subjects	ICC	LB	UB	*p*‐Value	ICC	LB	UB	*p*‐Value	ICC	LB	UB	*p*‐Value
10	21	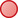 **.38**	−.05	.69	**.039**	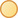 **.44**	.03	.73	**.019**	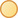 **.43**	.01	.72	**.022**
20	19	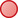 .36	−.10	.69	.058	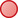 .34	−.13	.68	.074	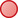 .15	−.32	.55	.269
30	19	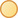 **.48**	.04	.76	**.017**	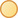 **.54**	.13	.80	**.007**	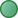 **.63**	.26	.84	**.001**
40	18	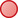 .34	−.14	.69	.080	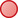 **.39**	−.08	.72	**.049**	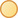 **.49**	.04	.77	**.017**
50	16	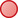 .36	−.14	.72	.076	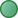 **.65**	.24	.86	**.002**	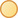 **.47**	−.02	.78	**.029**
all	23	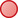 .29	−.13	.62	.082	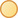 **.55**	.18	.78	**.003**	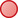 .27	−.15	.61	.104

*Note*: Colors represent the category of ICC: red for ICC values within the poor range, yellow for values within the fair range, and green for values in the good and excellent range. ICC and *p*‐values are printed in bold if they reach significance (*p*‐value < .05).

Abbreviations: ICC, intraclass correlation; LB, lower bound; UB, upper bound of the 95% confidence intervals of the ICC.

#### 3.2.2 Test–retest reliability of ERPs during face processing

The ICC values for the measures during the processing of upright and inverted faces are presented in Table 4. For traditional measures around the N290 component in the included sample, reliability values for peak latency across different numbers of trials were low (.04 ≤ ICCs ≤ .43). ICC values for N290 peak amplitude reached fair levels of reliability for 30, 50, 60, 80, and all available trials (.42 ≤ ICCs ≤ .56), whereas values for N290 mean amplitude reached fair levels at 50, 80, and all available trials (ICC = .56, .51, and .54, respectively). For P400 mean amplitude the ICC values were fair or higher from 40 trials and higher, with some values reaching good levels of reliability (.48 ≤ ICCs ≤ .76). ICC values for the DTW measure during the N290 time window were within the poor reliability range (−.15 ≤ ICCs ≤ .35).

**TABLE 4:** ICC values for EEG key metrics during face processing

		Included sample																			
		N290 peak latency				N290 peak amplitude				N290 mean amplitude				P400 mean amplitude				DTW during N290 window			
Individual ERP based on *N* trials	Number of subjects	ICC	LB	UB	*p*‐Value	ICC	LB	UB	*p*‐Value	ICC	LB	UB	*p*‐Value	ICC	LB	UB	*p*‐Value	ICC	LB	UB	*p*‐Value
10	38	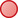 .04	−.28	.35	.407	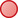 .27	−.05	.54	.050	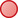 .14	−.19	.43	.205	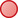 .23	−.09	.51	.077	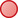 −.15	−.45	.17	.825
20	38	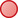 .25	−.07	.52	.064	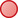 **.29**	−.02	.56	**.034**	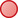 .27	−.05	.54	.050	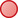 **.35**	.04	.60	**.013**	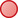 .09	−.24	.39	.303
30	36	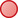 .21	−.12	.50	.105	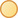 **.46**	.16	.69	**.002**	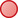 **.29**	−.04	.56	**.043**	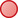 .19	−.14	.49	.126	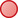 .25	−.08	.53	.069
40	33	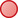 **.38**	.04	.63	**.014**	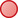 **.34**	.00	.61	**.026**	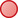 **.40**	.07	.65	**.010**	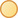 **.48**	.17	.70	**.002**	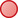 .13	−.22	.45	.236
50	32	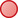 .11	−.24	.44	.268	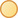 **.56**	.26	.76	**.000**	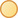 **.56**	.27	.76	**.000**	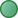 **.67**	.42	.82	**.000**	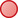 −.03	−.37	.31	.577
60	29	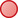 **.33**	−.04	.62	**.037**	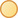 **.46**	.12	.70	**.005**	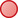 **.37**	.01	.64	**.023**	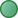 **.60**	.31	.79	**.000**	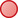 .30	−.07	.60	.054
70	27	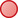 **.32**	−.06	.62	**.047**	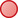 **.40**	.03	.67	**.018**	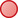 **.39**	.02	.67	**.021**	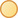 **.56**	.24	.77	**.001**	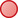 .15	−.23	.50	.219
80	25	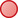 .32	−.08	.63	.055	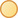 **.42**	.03	.69	**.017**	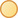 **.51**	.16	.75	**.004**	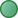 **.76**	.53	.89	**.000**	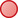 **.35**	−.04	.65	**.040**
90	22	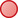 .34	−.09	.66	.058	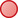 .32	−.11	.65	.070	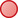 .31	−.12	.64	.078	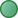 **.71**	.41	.87	**.000**	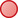 .06	−.37	.46	.400
100	21	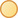 **.40**	−.02	.71	**.032**	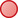 .22	−.23	.58	.168	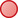 .24	−.21	.60	.143	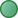 **.61**	.25	.82	**.001**	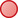 .33	−.11	.66	.066
all	38	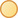 **.43**	.13	.65	**.003**	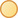 **.53**	.26	.73	**.000**	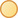 **.54**	.27	.73	**.000**	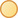 **.59**	.33	.76	**.000**	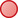 .10	−.22	.41	.267

		Highly attentive sample																			
		N290 peak latency				N290 peak amplitude				N290 mean amplitude				P400 mean amplitude				DTW during N290 window			
Individual ERP based on *N* trials	Number of subjects	ICC	LB	UB	*p*‐Value	ICC	LB	UB	*p*‐Value	ICC	LB	UB	*p*‐Value	ICC	LB	UB	*p*‐Value	ICC	LB	UB	*p*‐Value
10	23	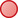 .22	−.20	.57	.153	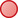 .19	−.24	.55	.191	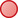 −.05	−.45	.36	.598	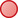 .02	−.38	.42	.456	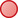 −.31	−.64	.11	.931
20	23	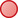 .31	−.11	.63	.073	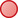 .11	−.31	.49	.308	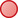 .11	−.31	.49	.311	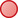 **.35**	−.06	.66	**.045**	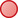 −.22	−.57	.21	.845
30	22	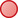 .16	−.27	.54	.232	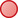 **.36**	−.06	.67	**.045**	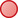 .07	−.35	.47	.373	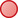 −.02	−.43	.40	.530	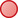 .06	−.37	.46	.399
40	21	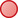 .22	−.22	.59	.160	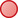 .21	−.24	.58	.176	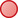 .32	−.12	.66	.072	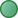 **.76**	.49	.89	**.000**	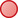 .08	−.35	.49	.361
50	20	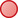 .10	−.35	.51	.328	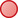 .33	−.12	.67	.071	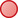 **.37**	−.07	.69	**.048**	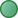 **.62**	.25	.83	**.001**	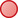 −.01	−.44	.43	.516
60	20	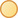 **.42**	−.02	.72	**.031**	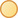 **.44**	.01	.74	**.022**	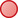 .32	−.13	.66	.075	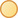 **.53**	.13	.78	**.007**	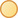 **.46**	.03	.74	**.019**
70	18	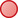 .20	−.28	.60	.207	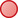 .34	−.14	.69	.080	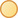 **.42**	−.05	.73	**.038**	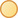 **.50**	.06	.78	**.014**	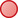 .18	−.30	.59	.233
80	18	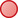 .28	−.20	.65	.123	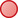 .33	−.15	.68	.087	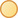 **.44**	−.02	.74	**.031**	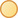 **.74**	.42	.89	**.000**	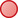 .28	−.20	.65	.123
90	17	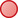 .22	−.27	.63	.186	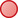 .31	−.18	.68	.104	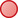 .30	−.20	.67	.113	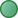 **.77**	.48	.91	**.000**	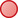 .05	−.43	.50	.427
100	17	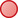 .36	−.13	.71	.069	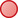 .21	−.29	.62	.205	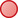 .22	−.28	.62	.195	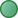 **.72**	.38	.89	**.000**	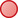 .31	−.19	.68	.108
all	23	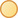 **.48**	.09	.74	**.009**	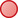 **.36**	−.05	.67	**.041**	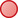 **.36**	−.05	.67	**.042**	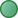 **.64**	.31	.83	**.000**	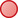 .23	−.19	.58	.140

*Note*: Colors represent the category of ICC: red for ICC values within the poor range, yellow for values within the fair range, and green for values in the good and excellent range. ICC and *p*‐values are printed in bold if they reach significance (*p*‐value < .05).

Abbreviations: ICC, intraclass correlation; LB, lower bound; UB, upper bound of the 95% confidence intervals of the ICC.

For the highly attentive sample, ICC values for N290 peak latency were mostly within the low range (.10 ≤ ICCs ≤ .48), similarly to the included sample. Reliability for the N290 peak amplitude and mean amplitude were more likely to be in the poor reliability ranges compared to the included sample (.11 ≤ ICCs ≤ .44 for peak amplitude, and −.05 ≤ ICCs ≤ .44 for mean amplitude). ICC values for P400 mean amplitude in the highly attentive sample reached fair and good levels of reliability from 40 trials and on (.50 ≤ ICCs ≤ .77), as in the included sample. The values for the DTW during the N290 window were within the poor range (−.31 ≤ ICCs ≤ .37), with the exception of DTW during the N290 window at 60 trials (ICC = .46).

#### 3.2.3 Test–retest reliability of face inversion effects

Table 5 displays the ICC values for the face inversion effects between the two visits across different numbers of trials included in the ERPs in the included sample and the highly attentive sample (see Table S1 for significance and direction of the face inversion effects). ICC values for the condition effects in the reliability sample were overall within the poor reliability range. For N290 peak latency and peak amplitude, ICC values ranged from −.24 to .32 and only the latter reached significance (for peak amplitude across 10 trials). ICCs for mean amplitude measures were also within the poor range (−.33 ≤ ICCs ≤ .36), with the exceptions of the measures across 60 trials with ICC = .55 for the N290 mean amplitude, and ICC = .49 for the P400 mean amplitude in the fair range. The same pattern was observed in the highly attentive sample where ICC values were within the poor range.

**TABLE 5:** ICC values for face inversion effect

		Included sample																			
		N290 peak latency				N290 peak amplitude				N290 mean amplitude				P400 mean amplitude				DTW during N290 window			
Individual ERP based on *N* trials	Number of subjects	ICC	LB	UB	*p*‐Value	ICC	LB	UB	*p*‐Value	ICC	LB	UB	*p*‐Value	ICC	LB	UB	*p*‐Value	ICC	LB	UB	*p*‐Value
10	38	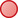 −.12	−.42	.20	.774	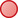 **.32**	.01	.58	**.023**	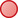 **.36**	.05	.61	**.012**	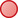 .24	−.09	.51	.075	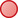 −.19	−.48	.13	.880
20	33	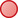 .28	−.06	.57	.054	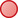 .20	−.15	.50	.134	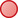 .23	−.12	.53	.094	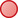 .18	−.17	.49	.160	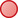 .13	−.22	.45	.230
30	29	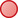 −.10	−.44	.28	.691	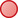 .09	−.28	.44	.319	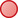 .04	−.33	.39	.419	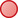 .05	−.32	.40	.406	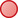 .20	−.17	.52	.144
40	25	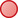 .07	−.33	.45	.368	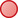 .01	−.38	.40	.483	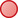 .02	−.37	.40	.470	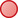 .02	−.37	.41	.459	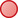 −.17	−.53	.23	.801
50	21	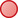 −.24	−.60	.21	.854	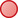 −.39	−.70	.04	.962	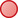 −.19	−.57	.25	.802	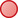 −.33	−.66	.11	.933	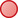 −.19	−.56	.26	.796
60	13	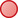 .08	−.47	.59	.388	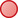 .24	−.33	.69	.199	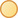 **.55**	.03	.84	**.020**	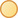 **.49**	−.05	.81	**.037**	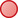 .16	−.41	.64	.294
all	38	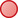 −.08	−.39	.24	.687	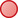 .04	−.28	.35	.402	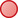 .17	−.15	.46	.148	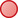 .08	−.24	.39	.310	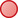 −.25	−.53	.07	.940

		Highly attentive sample																			
		N290 peak latency				N290 peak amplitude				N290 mean amplitude				P400 mean amplitude				DTW during N290 window			
Individual ERP based on *N* trials	Number of subjects	ICC	LB	UB	*p*‐Value	ICC	LB	UB	*p*‐Value	ICC	LB	UB	*p*‐Value	ICC	LB	UB	*p*‐Value	ICC	LB	UB	*p*‐Value
10	23	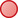 −.04	−.44	.37	.576	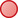 .18	−.24	.54	.201	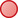 .23	−.19	.58	.141	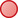 .03	−.38	.43	.440	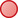 .24	−.18	.59	.127
20	21	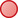 .21	−.24	.58	.178	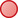 −.04	−.46	.39	.573	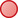 .04	−.39	.45	.430	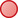 .02	−.40	.44	.460	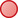 −.02	−.44	.41	.537
30	20	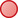 −.20	−.58	.26	.802	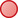 −.20	−.58	.26	.804	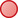 −.13	−.53	.32	.717	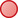 −.14	−.54	.31	.726	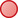 .37	−.07	.69	.048
40	18	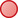 −.02	−.47	.44	.529	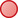 −.10	−.53	.38	.651	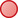 −.13	−.55	.35	.703	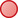 −.03	−.48	.43	.555	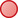 −.12	−.55	.35	.695
50	17	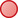 −.25	−.64	.25	.840	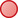 −.57	−.82	−.14	.994	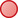 −.32	−.69	.17	.904	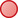 −.32	−.68	.18	.899	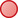 −.26	−.65	.24	.850
60	11	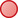 .08	−.52	.63	.399	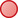 .20	−.42	.70	.264	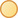 **.53**	−.07	.85	**.038**	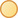 .48	−.13	.83	.056	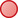 .29	−.35	.74	.184
all	23	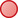 −.22	−.57	.21	.846	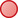 −.24	−.59	.18	.876	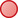 .03	−.38	.43	.453	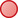 −.07	−.46	.35	.620	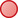 −.30	−.63	.12	.922

*Note*: Colors represent the category of ICC: red for ICC values within the poor range, yellow for values within the fair range, and green for values in the good and excellent range. ICC and *p*‐values are printed in bold if they reach significance (*p*‐value < .05).

Abbreviations: ICC, intraclass correlation; LB, lower bound; UB, upper bound of the 95% confidence intervals of the ICC.

### 3.3 Internal consistency of ERP measures

The test–retest reliability analyses (conducted across sessions 1–2 weeks apart) revealed ICC values that were mostly modest. We further examined the internal consistency of the ERP measures within each session by randomly drawing different numbers of clean trials from the datasets and splitting alternating trials into two datasets (A and B) for both the test and retest sessions separately. We then extracted each ERP component measure and calculated the internal consistency using the ICC between datasets A and B within sessions. The results for EEG key metrics during low‐level visual processing of checkerboards are presented in Table S2, and during face processing (faces upright and inverted collapsed) in Table S3.

The internal consistency for the checkerboards varied from poor to excellent (.03 ≤ ICCs ≤ .97) at both test and retest in the included sample, with a similar pattern in the highly attentive sample. Consistencies were good or excellent for P1 peak latency and DTW from 10 trials and above during the test session (.66 ≤ ICCs ≤ .97), but internal consistencies during retest session were fair and good from 20 trials or more (.53 ≤ ICCs ≤ .89). ICC values for P1 peak amplitude within sessions were fair from 20 trials during the test session (ICCs ≥ .40), but during the retest session these values were good at 30 trials (ICC = .64) and fair for all trials (ICC = .53). ICC values were within the poor range for lower trial numbers.

Internal consistency for the faces in the included sample also varied with most values being within the poor or moderate range, and a few reaching excellent reliability (−.08 ≤ ICCs ≤ .92). Internal consistency at the test session for N290 peak latency was moderate only for 60 trials and all trials (ICCs = .52 and .56, respectively), while values for N290 peak amplitude were moderate from 40 trials and above (ICCs ≥ .48). At the retest session, ICC values were higher with the moderate range for 30 and 50 trial for N290 peak latency, and from 20 trials and above for N290 peak amplitude (.41 ≤ ICCs ≤ .58), and even good at 60 for both N290 peak measures (ICCs = .64 and .92 for latency and amplitude, respectively). For N290 mean amplitude, internal consistency during the test session was poor across all trial numbers (ICCs ≤ .39), whereas at the retest session values were moderate from 20 trials and above (ICCs ≥ .48) and excellent values for 30 and 60 trials (ICCs = .67 and .90, respectively). Internal consistency for P400 mean amplitude was good for 40 and 50 trials during the test session (ICCs = .73 and .74) and excellent for 60 trials during the retest session (ICC = .80). Finally, internal consistency for DTW values during the N290 window was poor for all trial numbers at both test and retest session (−.08 ≤ ICCs ≤ .36), with the exception of internal consistency for 30 trials and 60 trials during the retest session (ICCs = .41 and .43). ICC values were within the poor range for all other trial numbers.

The highly attentive sample displayed slightly higher internal consistency values compared to the included sample during face processing. Several ICC values within the poor range for the included sample were within the fair range for the highly attentive sample: during the test session, for N290 peak latency across 10 and 20 trials at the test, for N290 mean amplitude across 40 and 60 trials, for P400 mean amplitude across 10 and 60 trials, and for DTW during the N290 time window across 40 trials (.41 ≤ ICCs ≤ .57); and during the retest session, for DTW during the N290 time window across all trials (ICC = .50). Other values moved from the fair range into the good and excellent range for the highly attentive sample: during the test session, for N290 peak amplitude across 40 and 60 trials; and during the retest session, for N290 peak amplitude across 30 and all trials, for N290 mean amplitude across all trials, for P400 mean amplitude across 30 and all trials, and DTW during the N290 time window across 30 trials (.60 ≤ ICCs ≤ .82). Finally, one ICC value in the highly attentive sample was within a lower range compared to the included sample: N290 peak latency across 30 trials (from ICC = .41 to .37). This suggests that measures of face processing may be more reliable in children who are more attentive to the screen.

## 4 DISCUSSION

This study set out to test the feasibility and reliability of an automated toolbox that included a gaze‐contingent stimulus presentation paradigm and an automated low‐density EEG processing pipeline for measuring event‐related responses to visual stimuli in toddlers.

### 4.1 Feasibility

We invited 61 children to take part in the study. Seven children (11%) refused to wear the EEG cap at the first session. This percentage is consistent with other reports where 11% of 5‐year‐old participants refused to wear the cap (Brooker et al., 2019). During the sessions, acceptability toward wearing the cap varied between toddlers. Some toddlers required more explanation and demonstration before agreeing to wear the cap, for example, parents or researchers themselves demonstrating how to wear the cap. It may be helpful to supply a video or storyboard demonstrating the capping procedure in a child‐friendly way to the families that they could watch prior to the visit. In addition, using age‐appropriate language and comparisons to everyday objects and actions may help children accept the cap more readily, for example: “the EEG gel is similar to daddy's hair gel,” “the EEG cap is like a hat,” and “you can do magic with your eyes and make the next picture appear on the screen.” It is good practice in development EEG research to take variability in EEG cap acceptance into account when designing and setting up an ERP study in preschoolers, particularly data loss due to cap refusal and flexibility in explaining the paradigm to the participants themselves.

Our reliability findings for visual processing of checkerboards suggest moderate reliability for the P1 component features when extracted from an ERP averaged across 20 trials or more. Based on this cutoff, we would be able to include 79% of the recruited participants in analyses on P1 peak latency and peak amplitude. We would be able to include 75% of the participants for the DTW measure in the P1 time window, using a cutoff of 30 trials with good reliability values. Our reliability findings for face processing indicate moderate reliability for the N290 peak amplitude measure can be reached with a minimum number of 30 trials per session. This would allow us to include 82% of the recruited participants. For N290 mean amplitude, we reached moderate reliability for 50 trials that would lead to the inclusion of 75% of our sample. Finally, reliability was moderate with a minimum of 40 trials for the P400 mean amplitude. This cutoff would leave 79% of the participants in the recruited sample being included in analyses. Taken together, these results suggest we can include 75%–82% of the recruited sample (or 88%–92% of the children with data at the first session without technical issues) depending on our research interests. These inclusion rates of 75%–82% from our study are higher than in previous research in toddlers (e.g., 50% in typically developing 18‐ to 30‐month olds (Webb et al., 2011)). These previous studies used a cutoff of 10 trials and did not focus on test–retest reliability, however. Inclusion rates in these studies may be even lower when they would focus on reliability. Overall, this indicates that our gaze‐controlled stimulus presentation paradigm with simultaneous low‐density, low‐cost EEG recording was successful in reducing dropout rate in toddlers.

In addition to dropout rates, we also examined the condition effects of face orientation during the face processing task. Our 2.5‐ to 4‐year olds did not display any differences in N290 ERP features between inverted and upright faces during early processing. These findings are consistent with studies reporting a lack of face inversion effects for the N170/N290 latency and amplitude in response to photographs of adult faces in 3‐year olds (Peykarjou et al., 2013) and schematic faces in 4‐year olds (Henderson et al., 2003). During later face processing, the P400 mean amplitude was larger for inverted than upright faces in the current study. A similar pattern was found in 3‐year olds (Peykarjou et al., 2013). The reliability of the face inversion effect for P400 mean amplitude reached modest values at 60 trials or more, suggesting 43% of the recruited sample may be included if one wants to examine individual differences in face inversion effects in this age group. It is possible that the pattern of face inversion effects for the N290 and P400 measures observed at this age is related to the pattern of reliability for these measures as discussed in the next section.

### 4.2 Reliability

The results of the test–retest reliability analyses revealed that reliability of the ERP components was overall moderate and was generally lower for latency than amplitude measures. For the P1 response to checkerboards, peak latency and amplitude showed fair reliability from 20 and 30 trials. Further, the DTW measure computed during the P1 time window (an alternative approach to determining latency) showed good reliability for 30 trials. For faces, reliability of N290 latency was poor across most numbers of trials, and DTW measures for faces showed poor reliability. This is unlikely due to the size of the time window as we visually inspected the peaks identified after the analyses and noticed peaks were correctly identified if their waveform had a clear shape. However, reliability for faces was fair for peak amplitude from 30 trials and for mean amplitude from 50 trials. Reliability for P400 mean amplitude was fair from 40 trials and good for higher numbers of trials. Thus, amplitude measures may be more reliable than latency measures of face processing in this age range. Furthermore, reliability of condition differences (face inversion effects) was generally within the poor range. Small or lack of face inversion effects at this age (Peykarjou et al., 2013) and the fact that face inversion measures are second‐order measures derived from first‐order measures of two conditions (Deuker et al., 2009) may contribute to the low reliability observed.

Overall, test–retest ICC values in our toddler sample were moderate. Few studies however have examined test–retest reliability in young children (Brooker et al., 2019) and none have examined how reliability varies for different numbers of trials included in the individual averages. One study in 10‐month‐old infants found ICC values of .76 for N290 mean amplitude (mean across a time window of 200–325 ms) and .56 for P400 mean amplitude (mean across a time window of 325–600 ms) for test–retest comparisons with a 2‐week interval and when all trials were included with a cutoff of 10 trials (Munsters et al., 2019). Reliability of the N290 peak latency was not tested in the study by Munsters et al. as not all infants showed a clear peak. Another study in 6‐11‐year‐old typically developing children revealed ICC = .80 for P1 amplitude and ICC = .66 for N170 latency for test–retest measures with a 6‐week interval (Webb et al., 2020). The ICC values in the current study are comparable with those for the P400 in the infant study but lower compared to the values in the study in older children. The lower values are possibly a result of lower signal to noise ratios in younger age groups compared to older age groups as found in studies focusing on ERPs during performance monitoring across childhood and adulthood (Hämmerer et al., 2013). Another possibility may be less stability in neural processing in younger age groups. Processes may stabilize more when they get more mature and intraindividual variability may decrease (Hämmerer et al., 2013). Indeed, our internal consistency analyses revealed higher ICC values for P1 measures than N290 and P400 measures suggesting higher signal to noise ratio and possibly more matured early‐stage perceptual processing compared to later‐stage face processing.

A range of factors may explain the differences in ICC values between measures and components in the current findings. First, the P1 peak is often more clearly detectable in individual ERPs than the N290 peak, as P1 peaks are sharper peaks with larger amplitudes while N290 peaks are often of wider with smaller amplitudes and may not be detectable in some individuals (Munsters et al., 2019). If selecting a measure to examine individual differences in the speed of early neural responses, P1 latency may be more reliable than the N290 latency and automated measures like DTW could provide a robust means of latency comparison. Second, mean amplitudes may be more reliable than peak amplitudes, which in turn may be more reliable than peak latencies. This is because noise overlaid on the signal will tend to be maximal at the peak, so averaging procedures across a time window will average out noise making mean amplitudes more reliable than peak amplitudes (Clayson et al., 2013). Third, differences in developmental stages may result in differences in reliability across neural responses during low‐level sensory processing and face processing. Low‐level sensory processing develops at a rapid pace during early postnatal development; the functional brain networks for visual processing show similar topographies to adult networks in neonates, whereas networks for higher order processing show similar topographies to adults during later postnatal periods at 1 or 2 years of age (Grayson & Fair, 2017; Keunen et al., 2017). Face processing in contrast has a protracted developmental trajectory that continues until the end of adolescence (Cohen Kadosh et al., 2013; Kilford et al., 2016). It is possible that individual differences in measures of low‐level visual processing are more stable compared to measures of face processing across the 2‐week interval during toddlerhood, as is also supported by our findings of higher internal consistency for low‐level visual processing measures than face processing measures within sessions.

Reliability values for DTW direction were overall lower than the values for the traditional ERP measures, for example, peak latency, peak amplitude, and mean amplitude. We included DTW in our measures because it has been proposed as a more robust measure of neural processing speed than peak latency (Zoumpoulaki et al., 2015). Here, we found that this measure might be more reliable for the P1 component during visual processing where there is a clearly detectable peak, but not for the N290 component where there is a less clearly detectable peak. Thus, DTW direction is less dependent on peak identification but requires a strong waveform morphology to function as a reliable and robust measure.

Our findings further suggested that sustained attention throughout the session did not affect reliability of low‐level processing measures because reliability values for P1 measures were similar across the included and highly attentive sample. In contrast, reliabilities for the N290 measures in the highly attentive sample were lower compared to those in the included sample but similar or even higher in the highly attentive than included sample for the P400 measure. Lack of differences between the included and highly attentive sample indicates that reliability does not worsen when including less attentive participants in general.

The characteristics of the FastERP task may further contribute to the reliability observed. The images of the checkerboard and four female faces were repeated throughout the paradigm. One possibility is that N290 features may be more reliable across trials with identical face stimuli. In the current analyses, we averaged across all artifact‐free face trials; thus, the number of trials included for each of the four female faces likely varies across analyses. Future work could examine whether responses may be more stable when only one face stimulus is included in the paradigm. Alternatively, one could argue that identical face stimuli may lead to habituation effects that may affect N290 and P400 responses (Itier & Taylor, 2004a; Jacques et al., 2007; Nordt et al., 2016; Schweinberger & Neumann, 2016); the reliability of N290 responses evoked from trial‐unique faces (Jones et al., 2016) is another option that could be explored in future work.

Analyses of the data have shown that the proposed gaze‐controlled paradigm provides moderately stable estimates of event‐related neural responses to checkerboards and faces during early development, comparable to those previously reported for infants (Munsters et al., 2019). As previously mentioned, use of videos or storyboards prior to the visit may improve acceptability of wearing the EEG cap among the young participants. We noticed in the lab that toddlers were able to complete the sessions due to the flexibility of the paradigm that allowed them to take brief, frequent and self‐determined breaks without the loss of data. The use of the wireless and mobile EEG system further facilitated this as toddlers could take a longer break away from the screen or even the room if needed and data collection would be paused (although this was rarely the case). Furthermore, real‐time analysis of the EEG data during the session may help ensure good data quality and prevent dropout due to artifacts. These suggested improvements may enable even greater flexibility for the participants and lower dropout rates in future developmental studies.

This study has important implications for the developmental field. First, the moderate reliability values suggest the gaze‐controlled paradigm and low‐density EEG processing pipeline may be suitable for developmental research. Second, the current findings are promising as low‐level and low‐density EEG systems are more scalable for use in the clinic and field (Lau‐Zhu et al., 2019). Further research will be needed to establish the suitability of the toolbox in LMIC populations and other age groups, for example, in toddlers in India or infants in The Gambia (http://braintools.bbk.ac.uk/), or children with neurodevelopmental disorders.

### 4.3 Limitations

We note that our paradigm has moderate test–retest reliability. Future work needs to explore whether other EEG features or other paradigms could achieve higher levels of reliability. Furthermore, this paradigm was designed for low‐density EEG systems. The advantages of these low‐density systems are their relatively low cost, scalability, portability, and potential for use outside of lab environment (Lau‐Zhu et al., 2019). Lab‐based EEG studies in developmental research have commonly used high‐density (HD) EEG systems with 32, 64, or 128 channels (e.g., in Munsters et al., 2019; Webb et al., 2020). Recording at a high number of EEG channels allows additional analyses such as connectivity (Bullmore & Sporns, 2009; van Wijk et al., 2010) or source localization of the brain signals measured in the developmental studies (Johnson et al., 2001). Future work may examine whether other recording systems may record signals achieving higher reliability. Future development of low‐cost, scalable HD systems will enable these measures to be brought into global EEG research.

## 5 CONCLUSION

In summary, we developed a novel toolbox with gaze‐controlled stimulus presentation and an automated preprocessing pipeline suitable for low‐density EEG systems that can be applied in large‐scale samples in field settings. We showed that the toolbox is feasible for use in visual processing research in toddlers, with inclusion rates of 79% for low‐level visual processing and 82% for face processing domains. Relevant to measures of individual differences, test–retest reliability over a 1‐ to 2‐week interval was moderate for a minimum of 20 and 30 trials for low‐level visual and face processing, respectively. Test–retest reliability and internal consistency of latency measures were higher for low‐level visual processing compared to face processing, whereas reliability and internal consistency for amplitude measures were similar or better during face processing compared to the low‐level visual processing. This suggests the speed and amplitude of low‐level visual processing and amplitude measures during face processing are relatively more stable over time, and thus may be more suitable measures of individual differences in visual perception/cognition in toddlerhood. The feasibility of automated and standardized solutions for data collection and analyses with low‐density EEG systems holds promise for large‐scale studies and application in global health.

## CONFLICT OF INTEREST

The authors declare no conflict of interest.

## AUTHOR CONTRIBUTIONS

**Rianne Haartsen**: Methodology, software, formal analysis, data curation, writing—original draft, visualization. **Luke Mason**: Conceptualization, methodology, software, data curation, writing—review and editing. **Eleanor K. Braithwaite**: Investigation, writing—review and editing. **Teresa Del Bianco**: Investigation, writing—review and editing. **Mark H. Johnson**: Conceptualization, writing—review and editing, supervision, funding acquisition. **Emily J. H. Jones**: Conceptualization, writing—review and editing, supervision, project administration, funding acquisition.

## Supporting information

Table S1

Table S2

Table S3

Supporting Information

## Data Availability

Scripts used for the gaze‐controlled stimulus presentation can be accessed through contacts on the Task Engine website (Jones et al., 2019; Mason, 2019). The scripts used for the EEG preprocessing and reliability analyses are available on GitHub (Haartsen et al., 2021). Data analyzed in the current study are available upon request via the BOND lab website (Del Bianco et al., 2019).
