# Supplementary material for: Reliability of an automated gaze‐controlled paradigm for capturing neural responses during visual and face processing in toddlerhood
Source: Dev Psychobiol. 2021 Sep 14;63(7):e22157. doi: 10.1002/dev.22157 (PMC9293026; doi:10.1002/dev.22157)
Supplement: Supplementary file 4 — Supporting Information [file DEV-63-0-s003.docx]

Supplementary Materials for:

Reliability of an automated gaze-controlled paradigm for capturing neural responses during visual and face processing in toddlerhood

SM1. Layout of the EEG cap

EEG was recorded at the channels Fpz, Fz, Cz, Oz, C3, C4, P7, and P8. Figure S1 displays a layout of the enobio system EEG cap.

**
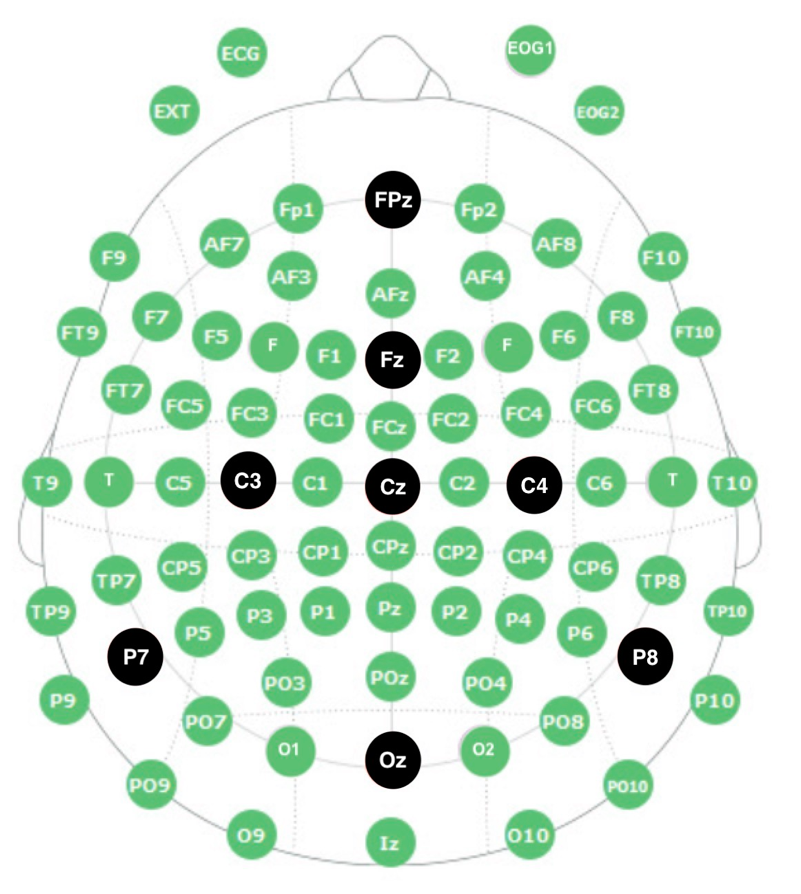
**

*Figure S1.* Layout for Enobio cap

The channels used in the Braintools paradigm are marked in black.

SM2. Exploring different threshold for EEG data cleaning

Previous studies have used a range of different thresholds as exclusion criteria for trials. These thresholds vary among labs and the ages of the participants whose EEG data is analysed. Due to this variation in the approaches, we decided to take a data-driven approach and explore how the use of different thresholds affects the quality of the individual ERPs for checkerboards and faces across all sessions with available EEG data.

To this end, we ran our preprocessing pipeline using the following thresholds: [-90, 90] μV, [-100, 100] μV, [-120, 120] μV, [-150, 150] μV, [-175, 175] μV, [-200, 200] μV, [-225, 225] μV, and [-250, 250] μV. For each session, we extracted the timeseries within the time window during stimulus presentation (0 – 500 ms) and calculated standard error of the mean (SEM) for each time point across the clean trials (Luck, Stewart, Simmons, & Rhemtulla, 2020). We then averaged these single point SEMs across the time window resulting in 1 single point single person SEM (spspSEM) value for each threshold for each session. Lower spspSEM values reflect better quality of EEG data.

In addition, we calculated the area under the curve (AUC) for the absolute timeseries during the baseline period (-100 – 0 ms) as a measure of data quality. As lower amplitudes during the baseline are indicative of better data quality, smaller areas under the curve reflect higher data quality. We finally also extracted the number of clean trials that would be available for each session with each threshold explored.

Results for the spspSEM and AUC analyses and numbers of clean trials in the checkerboard and face conditions are presented in Figure S2 and S3, respectively. The plots revealed that at the threshold of [-150, 150] μV the AUC across all session started to level off in both conditions while the number of included trials started to level off as well. The spspSEM values during the stimulus presentation seemed however relatively consistent across different thresholds. Based on the AUC and the trial numbers, we decided upon the threshold of [-150, 150] μV for further analysis.


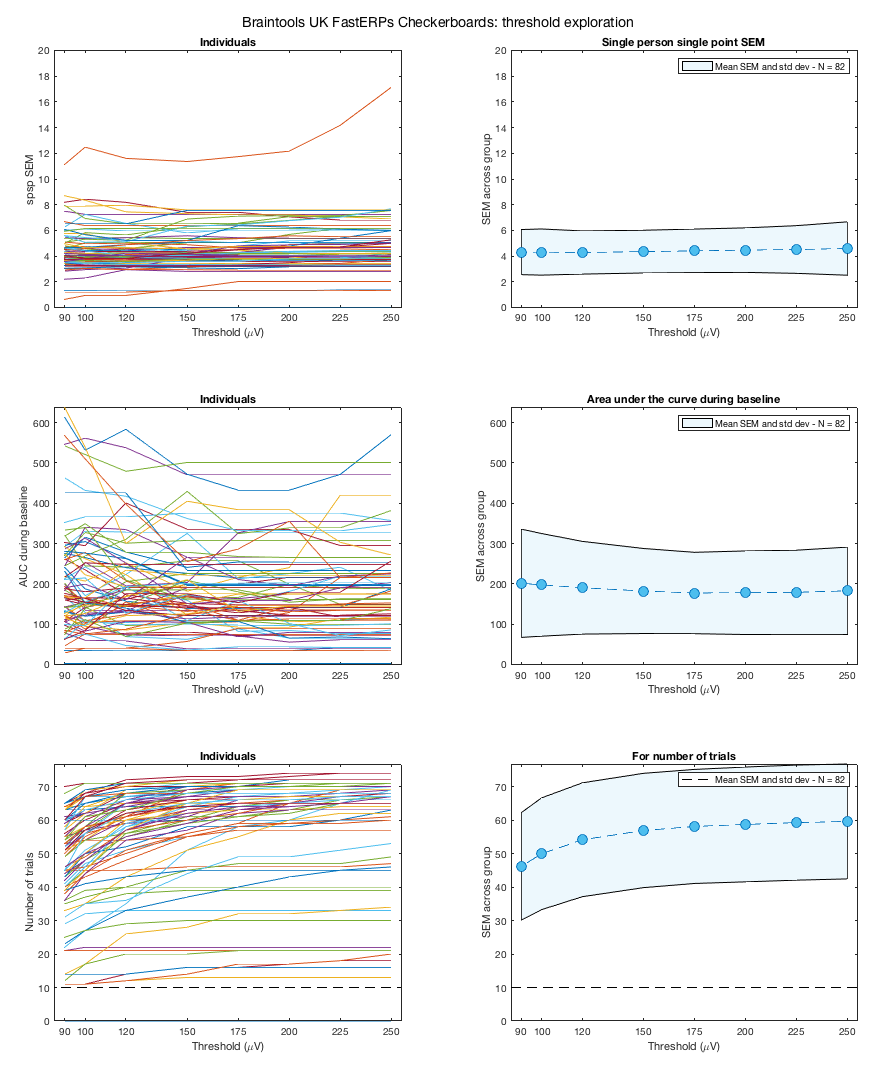


*Figure S2.* Exploration of thresholds for checkerboards

Data for individual session are plotted in the left column, while mean and standard deviation values are plotted in the right column. The top row displays values for the spspSEM measure, the middle row displays the area under the curve during baseline, and the bottom row displays the numbers or trials (dashed line marks 10 trials cut-off often used).


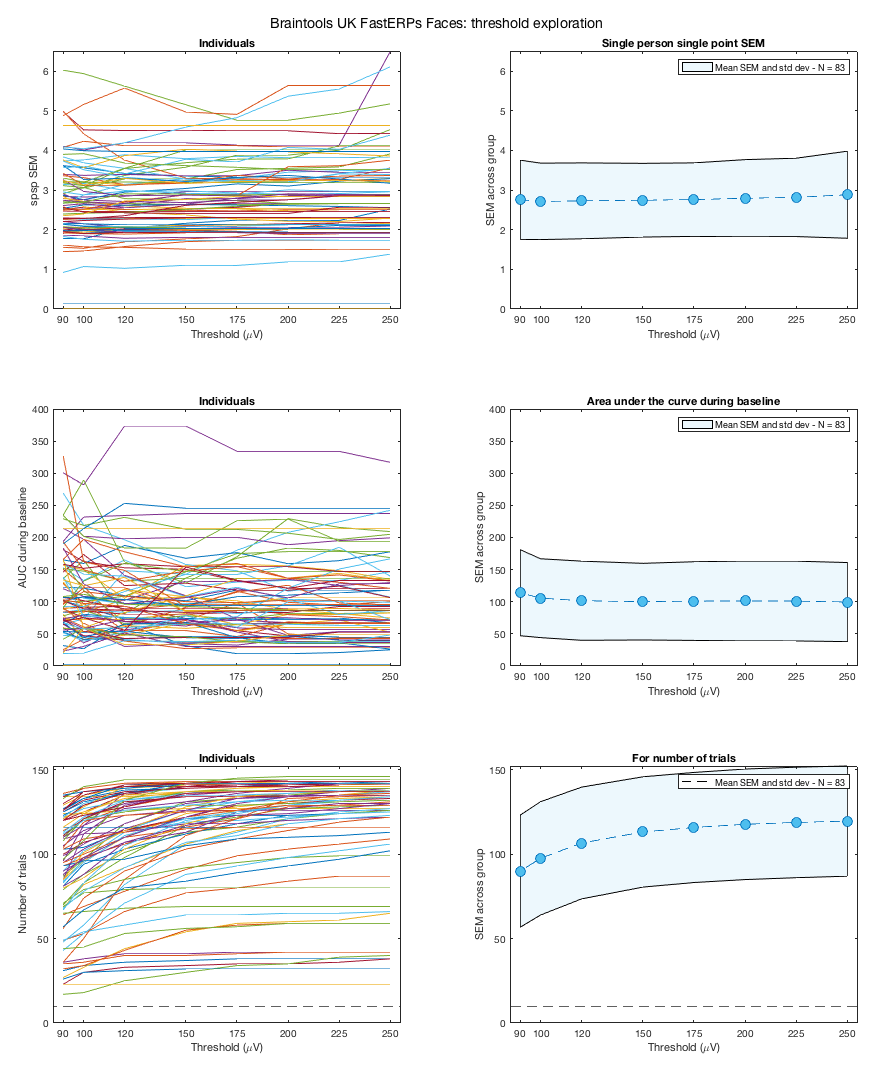


*Figure S3.* Exploration of thresholds for faces

Data for individual session are plotted in the left column, while mean and standard deviation values are plotted in the right column. The top row displays values for the spspSEM measure, the middle row displays the area under the curve during baseline, and the bottom row displays the numbers or trials (dashed line marks 10 trials cut-off often used).

SM3. Grand averages for the animal trials

The FastERP task included trials with face stimuli, checkerboards and animals. The animal trials were intended to help maintain the attention of the toddler participants during the session. For completeness, we also preprocessed the EEG data of the animal trials. The grand average for these trials is displayed in Figure S4 (plotted along with the grand averages displaying in figure 2c right in the main manuscript). Visual inspection of the grand average for animals reveals that the event-related responses here are relatively weak. Any ERP features extracted from these ERPs may not be comparable to the features extracted from the face and checkerboard ERPs. This is probably related to the toddlers counting or naming the animals and the low visual complexity in the animal images compared to the face and checkerboard images.


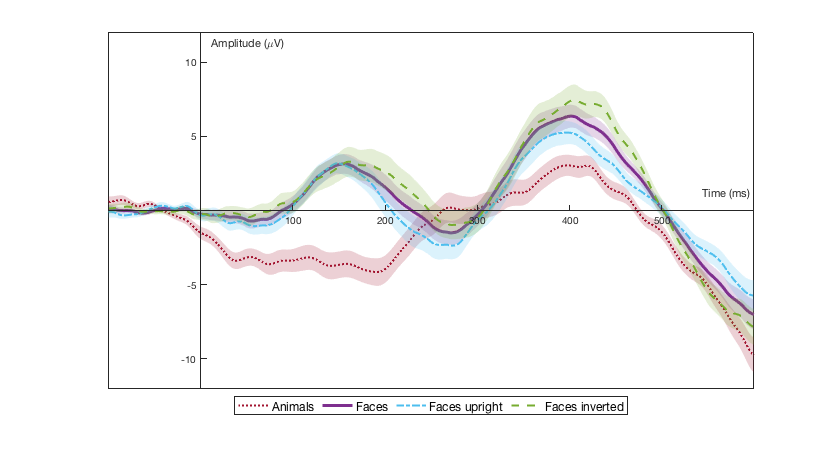


*Figure S4.* Grand averages for animals and faces

The grand averages across sessions for the animal trials, the face trials (collapsed across orientations), faces upright trials, and faces inverted trials. Shaded areas around the grand averages represent the standard error of the mean across sessions.

*References*

Luck, S. J., Stewart, A. X., Simmons, A. M., & Rhemtulla, M. (2020). Standardized Measurement Error: A Universal Measure of Data Quality for Averaged Event-Related Potentials (v20).
